# Regulation of β-Adrenergic Receptors in the Heart: A Review on Emerging Therapeutic Strategies for Heart Failure

**DOI:** 10.3390/cells13201674

**Published:** 2024-10-10

**Authors:** Warisara Parichatikanond, Ratchanee Duangrat, Hitoshi Kurose, Supachoke Mangmool

**Affiliations:** 1 Department of Pharmacology, Faculty of Pharmacy, Mahidol University, Bangkok 10400, Thailand; warisara.par@mahidol.ac.th; 2 Department of Pharmacology, Faculty of Science, Mahidol University, Bangkok 10400, Thailand; ratchanee.dun@student.mahidol.ac.th; 3 Pharmacology for Life Sciences, Graduate School of Pharmaceutical Sciences, Tokushima University, Tokushima 770-8505, Japan; hitoshikurose1436@gmail.com; 4 Pharmacology for Life Sciences, Graduate School of Biomedical Sciences, Tokushima University, Tokushima 770-8505, Japan; 5 Department of Pharmaceutical Care, Faculty of Pharmacy, Chiang Mai University, Chiang Mai 50200, Thailand

**Keywords:** β-adrenergic receptor (β-AR), β-arrestin, adenylyl cyclase (AC), CaMKII, cGMP, G protein, G protein-coupled receptor kinase, heart failure

## Abstract

The prolonged overstimulation of β-adrenergic receptors (β-ARs), a member of the G protein-coupled receptor (GPCR) family, causes abnormalities in the density and functionality of the receptor and contributes to cardiac dysfunctions, leading to the development and progression of heart diseases, especially heart failure (HF). Despite recent advancements in HF therapy, mortality and morbidity rates continue to be high. Treatment with β-AR antagonists (β-blockers) has improved clinical outcomes and reduced overall hospitalization and mortality rates. However, several barriers in the management of HF remain, providing opportunities to develop new strategies that focus on the functions and signal transduction of β-ARs involved in the pathogenesis of HF. As β-AR can signal through multiple pathways influenced by different receptor subtypes, expression levels, and signaling components such as G proteins, G protein-coupled receptor kinases (GRKs), β-arrestins, and downstream effectors, it presents a complex mechanism that could be targeted in HF management. In this narrative review, we focus on the regulation of β-ARs at the receptor, G protein, and effector loci, as well as their signal transductions in the physiology and pathophysiology of the heart. The discovery of potential ligands for β-AR that activate cardioprotective pathways while limiting off-target signaling is promising for the treatment of HF. However, applying findings from preclinical animal models to human patients faces several challenges, including species differences, the genetic variability of β-ARs, and the complexity and heterogeneity of humans. In this review, we also summarize recent updates and future research on the regulation of β-ARs in the molecular basis of HF and highlight potential therapeutic strategies for HF.

## 1. Introduction

β-adrenergic receptor (β-AR) is a member of the G protein-coupled receptor (GPCR) family that plays an essential role in the sympathetic nervous system (SNS). The overstimulation of β-AR contributes to cardiac abnormalities, resulting in the development of cardiac remodeling and the progression of heart failure (HF) which remains a major global health problem [1]. β-ARs are classified into four subtypes including β_1_-AR, β_2_-AR, β_3_-AR, and the putative β_4_-AR. While all the subtypes can couple to the Gαs protein and subsequently stimulate adenylyl cyclase (AC) to generate the second messenger 3′, 5′-cyclic adenosine monophosphate (cAMP), considerable differences exist in the ability for them to activate downstream effectors and their regulators [1,2]. Phosphodiesterases (PDEs) involve β-AR signaling by hydrolyzing cyclic guanosine monophosphate (cGMP) and cAMP [3]. In the heart, PDE isoforms like PDE3 and PDE4 finetune cAMP levels in specific compartments, ensuring precise signal transduction, which affects cardiac contractility and relaxation [4]. By preventing excessive cAMP accumulation, PDEs help avoid pathological conditions, maintaining a balance between β-AR stimulation and the appropriate cardiac response under stress or hormonal changes [3]. It is noteworthy that some of these conditions are characterized by the dysregulation of the SNS, resulting in enhanced β-AR stimulation [1,5]. However, the pathological consequences of the crosstalk between β-ARs and their regulators in the heart as well as the mechanisms by which β-AR stimulation is associated with the pathophysiology of heart diseases remain unclear.

In the heart, β_1_-AR is the predominant subtype, comprising 75–80% of the total β-AR density, while β_2_-AR accounts for 20–25%, and the remaining approximately 2–3% consists of β_3_-ARs in the normal heart [6]. β_2_-AR is more dominant in other types of cells, including vascular smooth muscle cells and endothelial cells [6]. β_3_-AR is also found in the hearts and serves as one of the therapeutic targets for the treatment of HF [7]. The existence of a putative fourth subtype, β_4_-AR, has been demonstrated by the results obtained with CGP-12177 (a partial agonist of β_3_-AR with β_1_-/β_2_-AR antagonist [8,9]; however, the physiological role of putative β_4_-AR in the heart remains poorly understood. Even though all the subtypes are well known as GPCRs that primarily couple with the Gαs protein, β-ARs display distinct and unique characteristics in signal transduction and intracellular responses. 

This review focuses exclusively on the regulation of β-ARs at the receptor, G proteins, and effector loci, as well as their signal transduction in the physiology and pathophysiology of the heart. We also provide insight into promising therapeutic targets for HF, focusing on the regulation of β-ARs in the heart, as well as the recent clinical studies of β_3_-AR agonist, AC6 gene therapy, G protein-coupled receptor kinase 2 (GRK2) inhibitors such as paroxetine and β-adrenergic receptor kinase carboxyl-terminus (β-ARKct), Ca^2+^/calmodulin-dependent protein kinase II (CaMKII), and sarcoplasmic/endoplasmic reticulum Ca^2+^-ATPase (SERCA2a) for the treatment of HF. The published literature on β-AR regulation in the heart and therapeutic strategies for HF was comprehensively searched in standard electronic databases such as PubMed, Embase, ScienceDirect, and Scopus. The search was carried out using relevant keywords including β-AR, β-arrestin, CaMKII, G protein, AC, GRK, and HF. For preclinical studies, the data encompass study design (in vitro or in vivo), β-AR subtype and regulation, study models and inducers, and key findings related to cardiac biomarkers. For clinical studies, the data include study design, clinical identifiers, condition and number of participants, interventions, inclusion criteria, and main clinical outcomes in terms of efficacy and safety.

## 2. Signal Transductions of β-ARs in the Regulation of Heart Functions

### 2.1. β_1_-AR Signaling

Norepinephrine (NE) is a major catecholamine secreted from the nerve endings of the SNS to regulate heart rate and cardiac contractility [10,11]. Epinephrine, also known as adrenaline, is released into the circulation from the adrenal medulla, which also regulates cardiac functions through β-ARs [10,11]. Although the binding affinities of both β_1_-AR and β_2_-AR to NE and epinephrine are quite similar, β_1_-AR has a higher binding affinity to NE and epinephrine than β_2_-AR [6,12]. After agonist binding, β_1_-AR couples with Gαs protein which subsequently stimulates AC activity, leading to cAMP production. Consequently, cAMP binds to and activates protein kinase A (PKA), which in turn phosphorylates several key proteins involved in the regulation of heart rate and contractility and induces positive chronotropic and inotropic effects which occur in conjunction with an increase in intracellular Ca^2+^ levels [2,6]. Overall, catecholamines play a significant role in regulating heart dynamics following the acute stimulation of β_1_-AR, which transduces signals through the Gαs/AC/cAMP/PKA pathway. In contrast, the chronic and excessive stimulation of β_1_-AR by catecholamines promotes adverse cardiac remodeling and HF [6,13]. Although cAMP mainly activates PKA, several lines of evidence suggest that it can also activate hyperpolarization-activated cyclic nucleotide-gated (HCN) channels, which are believed to play a vital role in the depolarization of cell membrane, resulting in the generation of action potential followed by cardiomyocyte contraction [14].

The activation of PKA is a key step for controlling the strength and duration of cardiomyocyte contraction after β_1_-AR stimulation. The activated PKA phosphorylated L-type Ca^2+^ channel leads to a Ca^2+^ influx and an increase in intracellular Ca^2+^ levels. This Ca^2+^ channel is opened during membrane depolarization and the generation of the action potential, which is essential for the initiation of cardiomyocyte contraction [15]. Moreover, PKA activates the ryanodine receptor (RyR), leading to a rapid elevation of intracellular Ca^2+^, improved sarcomeric shortening, and increased cardiac contraction force [16]. In addition to the Gαs protein-dependent pathway, β_1_-AR stimulation activates the epidermal growth factor receptor (EGFR) signal transduction in the heart via β-arrestins, a process known as receptor transactivation, resulting in cardioprotective effects [17].

### 2.2. β_2_-AR Signaling

The stimulation of β_2_-AR has been shown to have a similar impact as β_1_-AR, as it can increase the contractile force within the heart. For example, β_2_-ARs regulated L-type Ca^2+^ channels via a cAMP/PKA-dependent manner, thereby increasing cardiac contraction [6,13]. However, it is less potent than that of β_1_-AR stimulation. In addition to the Gαs/cAMP/PKA-dependent pathway, β_2_-AR transduces signals independently of PKA to activate Na^+^/H^+^ exchange regulatory factor (NHERF) [18]. So far, it was recognized that the signal transduction of β_2_-ARs specifically relied on a Gαs protein-dependent manner. However, it is now acceptable that β_2_-AR also transduces the signal through the Gαi protein [19]. The activation of β_2_-ARs possessed remarkable cardioprotective effects especially in the conditions of hypoxia and oxidative stress through the Gαi/PI3K/Akt signaling pathway [20]. In addition, β_2_-AR stimulation was found to have a significant role in EGFR transactivation via the PI3K-dependent manner [21].

Moreover, β_2_-AR activation inhibited phospholipase C epsilon (PLCε) at the Golgi apparatus through a mechanism that requires β_2_-AR internalization and extracellular signal-regulated kinase (ERK) signaling from endosomes. β_2_-AR stimulation in endosomes counteracted β_1_-AR-mediated cardiomyocyte hypertrophy, highlighting the cardioprotective effects of β_2_-AR [22]. The stimulation of β_2_-ARs resulted in an increased binding affinity of the Gαi protein to β_2_-AR through the PKA-mediated phosphorylation of the receptor, which serves as a protective mechanism of the heart against adverse conditions [23]. Additionally, it was reported that the stimulation of β_2_-ARs regulated p38 mitogen-activated protein kinases (p38 MAPKs) via Gαi protein-dependent fashion, and the induction of p38 MAPK exerted antiapoptotic effects by counteracting apoptotic events in adult rat cardiac myocytes [24]. However, the inhibition of p38 MAPK was unable to completely block the antiapoptotic effects of β_2_-ARs [25]. Therefore, other pathways such as ERK1/2 and Gβγ-dependent PI3K signaling took part in synergistic protecting cardiac cells against apoptosis.

In response to stimuli, several immune cells activate adrenergic receptors, mainly β_2_-ARs, leading to the initiation of a G protein-dependent signaling pathway that impacts a range of cellular functions and responses [26]. Signaling through β_2_-ARs is essential for the regulation of the immune response during injury, as it inhibits the release of inflammatory cytokines from macrophages and dendritic cells. This inhibition diminishes the overall responses and affects the behavior of these important immune cells during inflammation [27,28]. Besides inhibiting inflammatory cytokine secretion, β_2_-AR signaling also influences innate immune cell activities, including their activation, migration, and interactions, which in turn affect the activation, differentiation, and responses of both B and T lymphocytes, thus shaping the adaptive immune response [26]. Interestingly, both myofibroblasts and inflammatory cells play key roles in cardiac fibrosis, with myofibroblasts forming scar tissue and inflammatory cells releasing signals that enhance and perpetuate the fibrotic response [29].

Colchicine has been shown to enhance β_2_-AR-mediated vasodilation in arteries from spontaneously hypertensive rats [30] and in patients with essential hypertension [31], indicating that colchicine modulates β_2_-AR activity. However, its cardiovascular benefits are more likely due to its anti-inflammatory properties [32]. As β_2_-AR stimulation plays an essential role in immunomodulatory and anti-inflammatory activities, further studies are needed to investigate the mechanisms by which colchicine regulates β_2_-AR signaling during cardiac injury. Nevertheless, it is not yet fully elucidated whether β_2_-AR is responsible for recruiting inflammatory cells that regulate both physiological and pathological conditions.

### 2.3. β_3_-AR Signaling

Although the exact role of β_3_-AR in the heart remains unclear, a previous study demonstrated that β_3_-AR played a role in the activation of the L-type Ca^2+^ channel in atrial cardiomyocytes, contributing to an increase in atrial contractility [33]. In human ventricles, the stimulation of β_3_-AR did not increase the ventricular contractility, while it slightly increased L-type Ca^2+^ current [34]. These inotropic effects of β_3_-AR involved the cAMP/PKA-dependent signaling pathway, implying the coupling with Gαs protein. Apart from β_1_-AR and β_2_-AR, β_3_-AR exhibits distinctive coupling with both Gαs and Gαi proteins, influencing cardiac functions and remodeling [35,36]. Since β_3_-ARs are coupled with Gαi protein, they may serve as a regulatory mechanism to inhibit the overactivation of β_1_-ARs and β_2_-ARs [37].

In animal models and human heart tissues, it has been reported that β_3_-ARs stimulate nitric oxide synthase (NOS) activity, leading to the elevation of nitric oxide (NO) levels and the subsequent activation of downstream NO-sensitive guanylyl cyclase (NO-GC)/cGMP signaling pathway. This process partially accounts for the reduction in excitation–contraction coupling, eliciting the negative inotropic effects of β_3_-AR stimulation in healthy cardiomyocytes, while also offering protection to failing myocytes [38,39], highlighting the importance of the NOS/GC/cGMP pathway as a key mechanism of β_3_-AR signaling in addition to a well-known Gαs protein. In addition, β_3_-AR stimulation reversed oxidative modifications on the cardiac Na^+^-K^+^ pump, which reduced excess intracellular Na^+^ levels and enhanced excitation–contraction coupling and contractility in failing hearts [40]. Consequently, β_3_-AR agonists (such as mirabegron) are potential candidates for the treatment of HF.

### 2.4. Biased Signaling of β-ARs

Biased agonism is the ability of a GPCR to preferentially activate specific signaling pathways over others, leading to selective physiological responses. β-ARs can interact with various G proteins and β-arrestins through biased signaling, where ligands that stabilize specific receptor conformations selectively activate distinct signaling pathways and transducer subsets, leading to diverse outcomes [41]. Biased cellular responses can also arise from additional mechanisms, such as receptor bias, system bias, and location bias, which influence how receptors interact and signal within different contexts [41]. Receptor bias describes the tendency of a receptor to preferentially couple with one transducer over another, compared to a standard, due to its intrinsic properties. For instance, β_1_-ARs exemplify receptor bias as they are predominantly expressed in cardiomyocytes, whereas β_2_-ARs and β_3_-ARs are primarily found in nonmyocytes, highlighting the variation in the distribution of β-AR subtypes [42].

Carvedilol shows β-arrestin-biased signaling at β_1_-ARs by activating β-arrestin over G proteins and triggers ERK signaling that protects the heart during ischemia/reperfusion by reducing damage and enhancing function [43,44]. In addition, the induction of β_1_-ARs is found to involve the recruitment of β-arrestins, which facilitates the activation of the EGFR pathway, and requires the presence of GRK5 and GRK6 to phosphorylate β_1_-AR and enables the subsequent signaling cascade [17]. In addition, carvedilol functions as a β-arrestin-biased agonist at the β_2_-AR while acting as an inverse agonist by inhibiting Gαs protein signaling, which differs from its effects at β_1_-AR [45]. These results highlight the variations in the downstream effectors of each β-AR subtype. Since bias selectively activates particular downstream signaling, biased ligands can promote beneficial effects while minimizing off-target interactions at a specific GPCR, which has made the development of biased agonists a vibrant and active field of research.

## 3. Role of β-ARs in Heart Failure (HF)

The failing heart can be triggered by the overactivation of the SNS from the stimulation of β_1_-ARs. Initially, this compensatory mechanism provided advantages for the heart by increasing heart rate and cardiac contraction [46,47]. However, prolonged and sustained β-AR stimulation can lead to cardiac injury and remodeling associated with several pathological conditions, resulting in the progression of HF [46,47]. Therefore, the therapeutic values of the inhibition of β-AR signaling in HF remain an issue of clinical debate.

**Figure 1:**
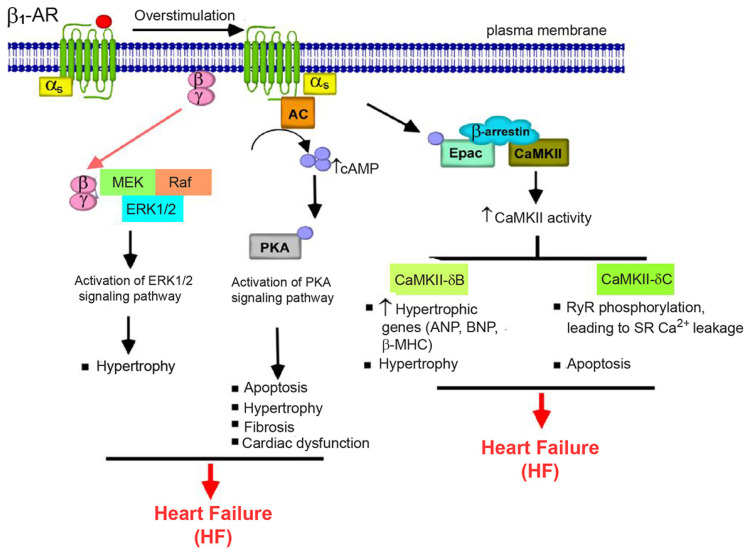
β_1_-AR signaling in heart failure (HF). The agonist stimulation of β_1_-ARs leads to the activation of AC via Gαs proteins, causing an elevation in cAMP levels. This cAMP, in turn, activates PKA and Epac and their downstream signaling pathways, resulting in cardiac hypertrophy, apoptosis, and fibrosis, leading to the development of HF.

The abnormality of β-ARs in terms of functions and signal transduction has been identified in the pathology of various heart diseases, especially HF (Figure 1). In patients with HF, there was a significant decrease in β_1_-AR expression together with an increase in receptor desensitization [47,48]. Moreover, the capacity of both β_1_-AR and β_2_-AR to activate and couple with their G proteins can be affected by persistent exposure to circulating catecholamines during failing hearts [10,11]. Transgenic mice with cardiac-specific overexpressed Gαs proteins revealed an enhancement of β-AR signaling through the Gαs/AC axis, resulting in initially improving cardiac functions but eventually causing long-term damage to the heart [49].

In addition, cAMP binds to and stimulates the exchange protein directly activated by cAMP (Epac) activity and its signal transduction, which is independent of PKA activation [50]. In response to chronic β-AR stimulation, Epac mediates the hypertrophic signaling pathway in the heart, involving several downstream effectors such as CaMKII, PLC, the small G protein H-RAS, and histone deacetylase type 4 (HDAC4). For instance, β-AR-mediated cAMP elevation triggers the activity of CaMKII isoforms, including CaMKII-δB and CaMKII-δC, which are located in the nucleus and cytosol, respectively [51,52]. CaMKII-δB increased the synthesis of hypertrophic proteins such as atrial natriuretic peptide (ANP), myosin heavy chain beta (β-MHC), and brain natriuretic peptide (BNP), while CaMKII-δC induced myocyte apoptosis and accelerated Ca^2+^ released from the sarcoplasmic reticulum, contributing to heart dysfunction [53]. Furthermore, Gβγ dimer which dissociated from Gαs protein activated the ERK1/2-dependent signaling pathway, leading to cardiac hypertrophy [54]. Another protein, synapse-associated protein 97 (SAP97), is a multifunctional scaffolding protein that interacts with the carboxyl terminus of the β_1_-AR and plays a significant role in the cardiotoxic β_1_-AR-CaMKII signaling in failing heart [55]. In mice with a cardiac-specific deletion of SAP97, β_1_-AR-mediated CaMKII activation occurs in an Epac-dependent manner, suggesting that the loss of SAP97 function results in the detrimental functional and structural remodeling of the heart [55]. Thus, the β_1_-AR-SAP97 signaling complex is reduced in HF.

Moreover, the stimulation of Epac resulted in the activation of the small G protein H-Ras, which is dependent on PLC and intracellular Ca^2+^ elevation in cultured cardiomyocytes. The activation of Epac also induced HDAC4 translocation via H-Ras-dependent signaling, highlighting the involvement of several Epac effectors in cardiac hypertrophy [56]. The β-AR-induced cardiac hypertrophy has been reported to be suppressed by the knockdown of Epac1 [57] and inhibition of Epac1 activity using AM-001, a thieno [2,3-b]pyridine derivative, identified as a specific Epac1 inhibitor [58]. Treatment with AM-001 reduced cardiac fibrosis and inflammation and improved cardiac functions during chronic β-AR stimulation in isoproterenol-treated mice [58]. Interestingly, Epac plays a crucial role in the β-AR switching process and in mediating differential effects on cardiac hypertrophy. Epac1 competed with PDE4D5 for interaction with β-arrestin-2 following β_2_-AR stimulation. The dissociation of the PDE4D5-β-arrestin-2 complex allowed Epac1 to be recruited to β_2_-AR, inducing a switch from β_2_-AR non-hypertrophic signaling to β_1_-AR hypertrophic signaling [59].

The overexpression of β_1_-ARs in the heart led to myocardial hypertrophy and fibrosis. Furthermore, the overstimulation of β_1_-ARs exhibited a significant decrease in cardiac contractility along with an increase in ages [60,61]. Moreover, hemodynamic investigations in transgenic mice demonstrated that cardiac contractility was initially enhanced, but later, less systolic and diastolic functions were seen, followed by an impairment of left ventricular (LV) contractility and relaxation [62]. As a result, prolonged β_1_-AR stimulation is one of the main etiologies of HF. Although the disadvantages of overexpressed β_1_-ARs in a healthy heart are well documented, more research is required to fully understand the benefits and drawbacks of altering β_1_-AR levels in the heart. Transgenic mice with the cardiac-specific overexpression of β_1_-AR or Gαs protein exhibited temporarily enhanced cardiac performance, but these effects ultimately accelerated the progression of HF [49,60]. However, aging-induced HF did not appear to have significant effects on β_2_-AR expression. Arrhythmia and increased mortality were also noted in transgenic mice with Gαs protein overexpression [49,63]. Furthermore, cardiac adverse effects are found in the heart with the excessive expression or prolonged stimulation of β_2_-ARs [64]. For these reasons, the excessive and persistent stimulation of either β_1_-ARs or β_2_-ARs can eventually cause dilated cardiomyopathy and HF.

In non-ischemic HF patients, the prevalence of insulin resistance is associated with HF progression [65]. Moreover, the condition of insulin resistance reduced the metabolic efficiency of cardiac muscle, resulting in contractile dysfunction [66,67], emphasizing the relationship between insulin resistance and heart diseases. The prolonged activation of β-ARs induced a state of insulin resistance in many tissues including the heart. Cardiac insulin resistance might be related to β-AR overstimulation and excessive SNS activation, leading to structural and functional abnormalities in the heart [68]. The excessive activation of β_2_-ARs attenuated insulin actions by reducing glucose uptake and glucose transporter type-4 (GLUT4) synthesis and translocation in cardiomyocytes and heart tissues [69]. In addition, the sustained stimulation of β_2_-ARs delayed GLUT4 translocation whereas short-term stimulation did not interfere in H9c2 cardiomyoblasts [70], indicating that cardiac insulin resistance appears to be predominantly mediated by prolonged β_2_-AR stimulation.

Interestingly, failing hearts have been shown to increase β_3_-AR mRNA and protein levels, and higher β_3_-AR expression has been observed in patients with HF [35]. The occurrence of cardiac hypertrophy and fibrosis in response to harmful stimuli was prevented in transgenic mice with the cardiac-specific overexpression of human β_3_-ARs [71]. Some evidence reported that the activation of β_3_-AR offered a protective effect on the heart after ischemia/reperfusion due to an increase in the NO levels, resulting from the upregulation of endothelial NOS (eNOS) and neuronal NOS (nNOS) [72]. The elevation of NO levels then triggered a downstream NO-GC/cGMP signaling pathway, leading to myocardium relaxation [35,38]. Recently, a clinical study investigating the effects of the β_3_-AR agonist mirabegron in HF patients found that after one week of regimen, mirabegron significantly increased cardiac index and decreased pulmonary vascular resistance in patients with moderate to severe HF with reduced ejection fraction (HFrEF) [73].

Currently, numerous studies attempt to explain the differences in signal transduction and regulation among β-AR subtypes. A deeper understanding of the functions and mechanisms of each β-AR subtype could provide valuable insights into developing therapeutic strategies to address cardiac pathologies, including hypertrophy, fibrosis, remodeling, and degeneration resulting from the prolonged overstimulation of β-ARs.

### 3.1. Changes in β-AR Expression and Signaling Network in Failing Hearts

In a failing heart, chronic β-AR activation led to the desensitization of receptors, which further decreased receptor responsiveness. In response to catecholamine-induced β-AR overstimulation, there was a noticeable decrease in mRNA and the protein expression of β_1_-ARs, but no significant change was observed in β_2_-AR levels [48,74]. Consequently, the ratio of β_1_-AR to β_2_-AR was altered from the normal ratio at 80:20 to 60:40 or 50:50, which was the ratio seen in the HF condition [74], indicating that the degree of HF was correlated with a reduction in the density of β_1_-AR [75]. Although the levels of β_2_-ARs did not significantly change in failing hearts, β_2_-AR stimulation-induced cardiac abnormalities have been observed. For instance, the stimulation of β_2_-AR promoted Ca^2+^-induced premature ventricular contractions in the failing heart, indicating the arrhythmogenic remodeling of β_2_-AR signaling [76,77]. Thus, non-selective β-blockers have been shown to offer greater benefits compared to selective β_1_-AR antagonists in chronic HF [78]. Remarkably, patients with dilated cardiomyopathy demonstrated significant shifts in gene expression, with some genes upregulated and others downregulated, reflecting a complex genetic response likely impacting disease progression, particularly in pathways related to cardiac contractility, metabolism, immunity, and the extracellular matrix. Approximately 430 genes were discovered to be involved in the downstream effects of β_1_-AR signaling, especially those related to metabolic processes and their link to ventricular remodeling [79].

### 3.2. Role of β-AR Genetic Polymorphism in HF

The pathology of HF not only originates from a consequence of the functional alterations and signal transduction of β-AR but is also closely related to the genetic polymorphism of β-ARs [80]. Human genetic epidemiology studies reported that the genetic variant of β-ARs is a risk factor associated with heart diseases, especially HF [80,81]. The polymorphisms of human β_1_-AR were mostly observed at position 389 where the amino acid is converted from arginine (Arg) to glycine (Gly), including β_1_-AR-Arg389 and β_1_-AR-Gly389 alleles (Table 1). The susceptibility of β_1_-AR to its agonist was found to be enhanced in β_1_-AR-Arg389Gly allele, which is associated with an increased risk of myocardial infarction (MI) and HF [82,83]. A study in transgenic mice overexpressing β_1_-AR-Arg389 demonstrated enhanced cardiac contraction at the initial stage of receptor stimulation; however, cardiac performance subsequently declined, suggesting that the β_1_-AR-Arg389 allele may contribute to cardiac dysfunction and HF progression [84].

The human β_2_-AR gene has been found to have polymorphisms in at least three positions, including amino acid residues 16, 27, and 164. An overexpression of the β_2_-AR-Ile164 allele has been shown to impair the coupling of β_2_-AR to Gαs protein, thus inactivating the downstream Gαs protein-dependent signaling pathway [90]. In addition, the prognosis of HF with β_2_-AR polymorphism confirmed that the β_2_-AR-Ile164 allele has more defects in β_2_-AR signaling, causing cardiac harmful effects when compared to the β_2_-AR-Thr164 allele [89] (Table 1). Even though the β_2_-AR-Ile164 variant does not directly cause HF, patients with HF who carry the β_2_-AR-Ile164 allele are at higher risk of losing cardiac adaptive mechanisms during HF condition [89]. Despite the genetic polymorphism of β-AR being discovered many decades ago, its role remains unclear. Consequently, further research is required to understand the implications of personalized medicine involving β-blockers in HF therapy.

## 4. Role of G Protein and AC in HF

### 4.1. Gαs Protein

In contrast to an increase in Gαi protein expression, the level of Gαs proteins remains unchanged in failing human hearts [91]. Transgenic mice with cardiac-specific Gαs protein overexpression exhibited the enhancement of β-AR-mediated Gαs/AC/cAMP signal transduction, resulting in positive inotropic and chronotropic effects [49]. However, the prolonged activation of Gαs protein can lead to detrimental effects such as cardiomyocyte death, cardiac fibrosis, and hypertrophy [49]. Similar to β_1_-AR, gene therapy targeting Gαs proteins should focus on inhibiting Gαs protein signaling rather than increasing Gαs protein. Interestingly, peptide inhibitors specific to the Gαs protein suppressed Gαs protein-dependent signaling [92]. However, further clinical studies should be performed to confirm the efficacy of this peptide inhibitor.

### 4.2. Gαi Protein

Enhancing β_2_-AR signal transduction while suppressing β_1_-AR signaling through gene therapy in animals is a promising approach for the intervention of HF. A study using transgenic mice revealed that Gαi2 protein is important for delayed myocardial hypertrophy and increases survival rate when β_2_-AR is activated [93]. In addition, an increase in Gαi2 proteins might have shown the protective effects against β-AR overstimulation in failing hearts via Gαi2 protein, resulting in an inhibition of AC activity. Hence, the gene delivery of Gαi2 protein to the heart could limit the contraction of cardiomyocytes induced by β_1_-AR [94]. Additionally, elevated levels of Gαi2 protein could provide cardioprotective effects by boosting antiapoptotic responses through the activation of β_2_-AR/Gαi2 signaling [95]. Therefore, the upregulation of Gαi2 protein in a failing heart may be an adaptive mechanism to protect the heart from apoptosis.

Gene therapy targeting the Gαi2 protein holds substantial promise as a therapeutic approach for arrhythmias. Research has demonstrated that increased levels of Gαi2 protein in the atrioventricular (AV) node led to a prolonged AV conduction and a lower heart rate during atrial fibrillation without heart block [96], suggesting that Gαi2 protein acts as an inhibitor of β_1_-ARs like β-AR antagonists by suppressing β_1_-AR-mediated AC/cAMP signaling. However, the gene therapy of Gαi2 protein in the heart should be further studied for the cardioprotective effects as well as the inhibition of potential cardiotoxic effects from prolonged β_1_-AR stimulation.

### 4.3. Adenylyl Cyclase (AC)

AC is activated by Gαs protein and inhibited by Gαi protein. Upon the stimulation of AC by Gαs protein, the activated AC catalyzes the formation of cAMP from ATP. cAMP is an important second messenger of the β-AR signaling. In mammals, at least nine AC subtypes, AC1 to AC9, have been identified [97]. However, the unclear specificity of AC isoforms in HF pathogenesis underscores the need for further studies in animal models to explore the therapeutic potential of novel compounds that selectively inhibit each AC isoform in the heart.

The inhibition of AC5 and AC6 activities has currently been identified as a promising therapeutic approach for HF. In mice with overexpressed β_2_-ARs, the disruption of AC5 reduced mortality and provided cardioprotection against chronic pressure overload [98] or β-AR-induced cardiomyopathy [99]. Furthermore, the activation of AC6 led to improved LV systolic and diastolic functions, evidenced by a higher ejection fraction and an increased slope of the end-systolic pressure–volume relationship (ESPVR) in mice with ischemic injury [100]. In an acute MI model, transgenic mice with the cardiac overexpression of AC6 exhibited a lower fatality rate and improved LV contractile functions, characterized by reduced LV dilation and increased ejection fractions. Additionally, these effects were linked to elevated cAMP production, the increased phosphorylation of phospholamban, and enhanced SERCA2a Ca^2+^ affinity [101]. The beneficial impacts of AC6 were further confirmed by using a genetic murine model of cardiomyopathy. It was found that the overexpression of AC6 improved cardiac performance, boosted cAMP generation, reduced myocardial hypertrophy, and extended lifespan [102,103]. Later, a recombinant adenovirus encoding AC6 gene (Ad-CMV-AC6) was employed for AC6 gene transfer in a normal pig through intracoronary infusion. After AC6 gene transfer, cardiac functions were improved as determined by increased LV contractility [104], highlighting the potential of AC6 as a potential target for HF therapy.

## 5. Role of G Protein-Coupled Receptor Kinases (GRKs) in HF

### 5.1. Introduction of GRKs

The GRK family includes seven members, GRK1 to GRK7, which can be divided into three subgroups based on their sequence homology of amino acid residues [105,106]. First, the rhodopsin kinase (visual GRK) subgroup consists of two members, GRK1 (rhodopsin kinase) and GRK7. GRK1 is predominant in the rod cells of the retina, whereas GRK7 is mainly detected in cone cells. Second, the β-adrenergic receptor kinase (β-ARK) subfamily comprises two members, GRK2 (β-ARK1) and GRK3 (β-ARK2). Third, the GRK4 subgroup includes three members, GRK4, GRK5, and GRK6. GRK4 is extensively distributed in the testes, cerebellum, and kidney, whereas GRK5 and GRK6 are abundantly expressed in several tissues [107].

In the human heart, GRK2, GRK3, and GRK5 are the major types of GRK family that were detected, whereas GRK4, GRK6, and GRK7 are rarely found [108,109]. GRK2 is expressed in cardiac myocytes and nonmyocyte cardiac cells, while GRK3 is abundantly expressed in cardiomyocytes. GRK5 is equally distributed throughout the heart [110]. Although the three GRKs most found in the heart are unique in their functions and signaling due to their distribution in cardiac cells, each type of GRK shares some common characteristics, for instance, their functions in receptor phosphorylation.

### 5.2. Pathophysiology of GRK in the Heart

In human cardiac tissue, the coupling and activation of Gαs proteins by agonist-occupied β-ARs as well as their signaling are impaired in failing hearts. This abnormality presumably results from an increase in both the production and activity of GRKs, especially GRK2, resulting in the desensitization and downregulation of β-ARs [109]. In addition, increased GRK2 levels and activity were found to be connected with the pathophysiology of HF and hypertension [111,112], supporting the pivotal role of GRK2 in cardiovascular diseases. Interestingly, GRK2 and GRK5 protein levels have been extensively raised in the isolated heart from the animal models of HF [110,113] and in the hearts of patients with dilated cardiomyopathy [114]. Furthermore, GRK3 levels were unaltered in the hearts of patients with dilated cardiomyopathy and slightly increased in patients with congestive heart failure [110,111,114]. Transgenic mice with GRK2 cardiac-specific overexpression exhibited an increase in GRK2 synthesis and activity in the heart, which led to the impairment of β-AR signaling and eventually contributed to myocardial hypertrophy [115]. To sum up, the upregulation of GRK2 expression and activity in the heart is an essential cause of HF. Therefore, the role of GRK2 on cardiac functions and its potential for the treatment of HF has been widely studied and GRK2 could be used as an early biomarker in HF.

The GRK5 levels in the heart were found to be unchanged in patients with HF, unlike animal subjects whose GRK5 content was increased in various models of HF such as cardiomyopathic hamsters [116], pacing-induced HF [117], and congestive HF in rats [110]. Interestingly, the cardiac-specific deletion of GRK5 prevented the dissociation of SAP97 from the C-terminus of β_1_-AR induced by agonists and led to increased CaMKII activity in the heart [55]. Therefore, GRK5 is essential for the dissociation of SAP97 from the β_1_-AR complex and facilitates the transition of β_1_-AR signaling to the Epac-dependent activation of CaMKII, which contributes to cardiac dysfunctions and the development of HF [55]. These data indicate the novel role of GRK5 in inhibiting the detrimental effects of β_1_-AR/CaMKII signaling. Collectively, the upregulation of GRK activity and synthesis in the heart is involved in the loss of β-AR signaling and functions which induces the development of HF.

### 5.3. Therapeutic Targets for HF: Focusing on GRK2

GRK2 has been extensively studied for its functional role in the heart. β-ARs are targets for GRK-mediated phosphorylation and desensitization. The upregulation of GRK2 activity and synthesis in the heart is associated with the loss of β-AR functions which enhances harmful effects, leading to the progression of HF, whereas the blockade of GRK2 upregulation can restore cardiac functions [109,118]. From the study of transgenic mice with cardiac-specific GRK2 overexpression, it was found that an increased GRK2 level led to reduced ventricular contractile force and cardiac AC activity as well as β-AR signaling which represents a similar pattern in failing hearts [115]. Thus, GRK2 has been implicated in the pathology of HF, and therapeutic strategies using the inhibition of GRK activity and production are promising targets for the treatment of HF [119].

#### 5.3.1. β-ARKct (or GRK2ct)

Both the activity and level of GRK2 are potentially inhibited by the overexpression of GRK2ct (β-ARKct) or gene ablation of GRK2, respectively (reviewed in [109,120]). β-ARKct is a peptide chain on the C-terminus of GRK2 containing 194 amino acids and a Gβγ binding domain is also found in the β-ARKct segment [115]. β-ARKct is used as a tool to selectively inhibit GRK2 activity, including GRK2-mediated β-AR desensitization. An overexpression of β-ARKct leads to an increase in the amount of β-ARKct, thereby competing with endogenous GRK2 for binding to the Gβγ subunit of β-AR (Table 2). Since GRK2 is unable to compete with the Gβγ subunit, the translocation of endogenous GRK2 to the cell membrane is suppressed and consequently inhibits GRK2-induced β-AR phosphorylation, resulting in a reduction in GRK2-mediated β-AR regulation, respectively [109,120].

In addition, the inhibition of GRK2 has been shown to reverse cardiac abnormalities in animal models of HF, for example, calsequestrin-overexpression mice [122] and muscle LIM protein knockout mice [121]. In fact, β-ARKct can normalize β-AR functions and restore ventricular functions as well as enhance LV performance [115]. In transgenic mice with β-ARKct overexpression, cardiac contraction was shown to improve under normal and β-AR agonist-induced contractions [115], highlighting the important role of GRK2 in cardiac function, particularly in cardiac contractility. Additionally, β-ARKct reduced the incidence of cardiac remodeling by inhibiting myocardial hypertrophy and increased survival rates [124]. Thus, β-ARKct gene therapy using adenovirus for gene delivery into the heart tissue has been applied to large animal HF models, which is likely to be an important strategy for HF therapy.

#### 5.3.2. Small Molecule Inhibitors of GRKs

Beyond employing the β-ARKct peptide, efforts have also focused on developing and screening small molecules that specifically inhibit GRK2 activity. For instance, paroxetine, a selective serotonin reuptake inhibitor (SSRI), binds to and inhibits the catalytic activity of GRK2 [125]. Many GRK2 inhibitors were also synthesized and found to have potent inhibitory effects on GRK2 activity such as GSK180736A and GSK2163632A [120]. Interestingly, the efficacy of paroxetine in patients with acute MI has been investigated [126,127] and the clinical outcomes of these studies will be discussed in Section 7.2. However, the efficacy of other synthetic GRK2 inhibitors has not been proven in preclinical and clinical studies. Furthermore, targeting GRK5 activity presents a novel approach to managing HF [128]; for example, strategies include the use of GRK5-specific small molecule interfering RNA (RNAi) delivered to the heart via adenovirus or the application of a specific peptide inhibitor to obstruct GRK5 activity within the heart. Nevertheless, the impact of GRK5 on cardiac functions in both normal subjects and HF patients needs to be further investigated.

## 6. Roles of β-Arrestins in HF

### 6.1. Introduction of β-Arrestins

Arrestin is one of the protein families that acts together with GRK for the desensitization and internalization of GPCRs. The interaction between arrestins and receptors results in a disconnection of receptor and G protein (uncoupling), thereby inhibiting G protein-dependent signal transduction. For this reason, this group of proteins is called “arrestin” from the word “arrest”. Arrestins comprise four members including visual arrestin, cone arrestin, β-arrestin-1, and β-arrestin-2. According to the similarity of amino acid sequences, functions, and distribution in the tissues, all four types of arrestins are divided into two main groups [105,129]. The first group consists of the visual arrestin and cone arrestin. Visual arrestin is the predominant protein located at the rod outer segment mainly found in the retina, while cone arrestin is abundantly located in the cone cells of the retina and the pineal gland [105]. The second group, β-arrestins, includes β-arrestin1 and β-arrestin2, both of which are found in several tissues, including the heart [105,129].

### 6.2. Cardioprotective Effects of β-Arrestins

β-Arrestin-mediated signaling by the β_1_-AR has led to the discovery of EGFR transactivation as a mechanism for β-arrestin-induced ERK activity following catecholamine stimulation [17]. Following phosphorylation by GRK5 or GRK6, the phosphorylated β_1_-AR can activate EGFRs in a β-arrestin-dependent process known as “transactivation” [17]. β-Arrestins have been reported to facilitate mitogenic ERK1/2 signaling by scaffolding Raf, mitogen-activated protein kinase kinase 1 (MEK1), and c-Src (a non-receptor tyrosine kinase) after β-AR stimulation. A variety of receptors, including β_1-_ARs [17] and β_2_-ARs [130], have been shown to activate ERK signaling in a G protein-independent, β-arrestin-dependent manner. Similarly, for β_2_-AR, the inverse agonist ICI118551 induces ERK phosphorylation that depends on β-arrestin-2 [131]. β-Arrestin2 delayed inflammatory responses by inhibiting the recruitment of macrophages to the infarcted size, leading to the suppression of inflammation and apoptosis in post-MI conditions [132]. Furthermore, β-arrestin2 has been demonstrated to prevent apoptosis [133,134], indicating the protective effects of β-arrestin2.

In addition, β-arrestin-1 can be phosphorylated at serine residue 330 (Ser330) by AMP-activated protein kinase (AMPK), as reported in both in vitro and in vivo studies, and this phosphorylation inhibits β-AR/cAMP/PKA signaling by increasing PDE4 expression and activity in the heart [135]. The phosphorylation of β-arrestin-1 at Ser330 also reduced isoproterenol-induced oxidative stress and inflammation in neonatal mouse cardiomyocytes. Mice with site-specific mutagenesis, knock-in with β-arrestin-1 S330D (the active form), demonstrated reduced isoproterenol-induced inflammation and myocardial fibrosis, revealing that the phosphorylation of β-arrestin-1 at Ser330 by AMPK exerts anti-inflammatory and antifibrotic effects by inhibiting the β-AR/cAMP/PKA pathway [135]. Thus, these findings support the idea that the catecholamine stimulation of cardiac β_1_-AR activates two distinct pathways, (1) a potentially harmful G protein-mediated pathway and (2) a protective β-arrestin-mediated pathway, which promotes EGFR transactivation, and attenuates apoptosis, inflammation, and fibrosis.

### 6.3. Therapeutic Targets for HF: Focusing on β-Arrestins

β-Arrestin-biased ligands are a group of ligands that can antagonize the G protein-dependent pathway of GPCRs and simultaneously activate signal transduction through the G protein-independent, β-arrestin-dependent pathway [136]. In this scheme, β-AR antagonist (β-blocker) is defined as ligands that can bind to β-AR and have no intrinsic activity by blocking agonist-induced G protein activation. Some β-blockers that antagonize β-AR-induced G protein activation also act as an agonist by stimulating β-arrestin-dependent signaling [45,137] (Table 3). The identification of novel ligands for β-ARs that block G protein signaling while simultaneously promoting β-arrestin signaling could provide a promising therapeutic approach for HF.

## 7. Emerging Therapeutic Targets for HF Focusing on β-AR Regulation

### 7.1. Clinical Studies of β_3_-AR Agonist

The cardioprotective effect of β_3_-AR is mediated through the activation of eNOS and nNOS via coupling to Gαi protein, which produces NO that subsequently triggers cGMP signaling [37,38,39]. Mirabegron, a β_3_-AR agonist, was approved for the intervention of overactive bladder by increasing bladder capacity by stimulating β_3_-ARs, which causes the relaxation of detrusor smooth muscle in the urinary bladder during the storage phase [141]. Due to the improvement in systolic function observed after β_3_-AR stimulation in the heart, mirabegron has been investigated as a potential therapy for HF (Table 4).

To evaluate the efficacy and safety of mirabegron, the initial first-in-human phase II clinical trial (BEAT-HF trial; NCT01876433) was conducted in 70 patients with HFrEF, characterized by a left ventricular ejection fraction (LVEF) of less than 40% and classified as New York Heart Association (NYHA) functional class II–III [143]. The patients were randomly assigned to receive either mirabegron 300 mg/day or a placebo. At 6 months, the findings were disappointing, as no significant difference in LVEF changes (mean difference, +0.4%; *p* = 0.82) or any of the secondary endpoints (volumetric parameters, physical capacity, and electrocardiogram changes) was observed between the mirabegron and placebo groups. In an exploratory analysis, the mean LVEF in patients with more severe HF at baseline (LVEF less than 40%) was increased in patients who received mirabegron compared with those given placebo (mean difference, +5.5%; *p* < 0.03) [143], suggesting that the activation of Na^+^-K^+^ ATPase induced by β_3_-AR agonist could be more effective in the treatment of advanced stages of HF. Due to the limited number of patients in this trial, further clinical studies with a larger patient population are required.

The BEAT-HF-II trial (NCT03926754) is the phase II/III clinical study assessing the efficacy and safety of mirabegron, enrolling 22 HFrEF patients with LVEF of less than 35%, NYHA class III-IV, and increased N-terminal pro-B-type natriuretic peptide (NT-proBNP) levels, to evaluate the hemodynamic response to mirabegron [73]. Either mirabegron 300 mg/day or a placebo was randomized to the patients as an add-on to standard therapy. After one week of regimen, the mirabegron treatment led to a significant improvement in cardiac performance by increased cardiac index (mean difference, +0.41 L/min/BSA; *p* = 0.039) and reduced pulmonary vascular resistance (PVR) (−1.6 Wood units; *p* = 0.02), while no differences were observed in changes in the blood pressure, heart rate, and systemic vascular resistance (SVR) between the mirabegron and placebo groups. For the safety profile, mirabegron was well tolerated [73]. Therefore, mirabegron represents a potential therapeutic target for the treatment and prevention of HF. However, the efficacy and tolerability of mirabegron need to be confirmed through clinical trials involving longer durations and larger numbers of HF patients.

LV hypertrophy and diastolic dysfunction play a role in the onset and progression of HF, especially in patients at the early stages of the disease. The phase IIb clinical trial (Beta3-LVH trial; NCT02599480) included 296 patients with or without HF symptoms (NYHA class I–II), who had LV hypertrophy defined as an increased LV mass index (LVMI) (men ≥ 115 g/m^2^ or women ≥ 95 g/m^2^) or a maximum wall thickness greater than 13 mm [142]. The participants were allocated to receive mirabegron (50 mg/day) or a placebo for 12 months. At the endpoint, the adjusted differences between the groups, accounting for baseline and covariates, showed an increase in LVMI (+1.3 g/m^2^; *p* = 0.08) and a decrease in the early diastolic tissue Doppler velocity [E/e’] ratio (−0.15; *p* = 0.60). Mirabegron was generally well tolerated [142]. Overall, the mirabegron therapy had no impact on preventing HF in the patients with structural heart disease who presented with no or mild HF symptoms.

Pulmonary hypertension (PH) is a common complication of HF and is associated with a high burden of morbidity and poor prognosis. The SPHERE-HF trial (NCT02775539) is a recent clinical trial performed in 80 stable patients with combined pre- and post-capillary PH (CpcPH) associated with symptomatic HF to assess the potential benefits of mirabegron in improving clinical outcomes [144]. The patients were assigned to either mirabegron (200 mg/day) or a placebo for 16 weeks. However, the findings indicated that there was no significant achievement of the alteration in PVR as measured by right heart catheterization (mean difference, +0.62 Wood units, *p* = 0.218). The patients treated with mirabegron showed an improvement in right ventricular ejection fraction compared to those receiving placebo (mean difference, +3.0%, *p* = 0.026), although no differences were observed in the other secondary outcomes, including hemodynamic parameters, functional class, and quality of life [144]. Although the potential benefit of β_3_-AR agonist in PH was found to be negative, further research is needed to explore their effects.

In summary, despite showing negative results on key clinical measures, especially LVEF in patients with HFrEF, the β_3_-AR agonist remains a promising therapeutic option for the treatment of HF due to its beneficial effects on the other parameters such as increased cardiac index and improved cardiac performance as well as the favorable safety (Table 4). Given its good tolerability under full β_1_/β_2_-AR blockade in HFrEF patients, this is encouraging for individuals who are already using mirabegron for urological conditions. However, to apply these findings to standard clinical practice for the management of HF, further validation is required through additional phase II/III clinical trials with a longer duration and a larger patient population.

### 7.2. Clinical Studies of GRK2 Inhibitor

Given the complex role of GRK2 in cardiovascular regulation, ongoing research aims to identify specific inhibitors that can effectively target this kinase without causing adverse effects. The development of GRK2 inhibitors for heart disease is an area of active investigation with the potential to offer new treatment options for patients with this debilitating condition. Paroxetine is being explored for its off-target effect in reversing cardiac remodeling and improving LVEF as a potential therapeutic option for managing acute MI, with clinical trials underway to assess its safety and efficacy (Table 5).

The most recent study, known as the CARE-AMI trial (NCT03274752), evaluates the efficacy of paroxetine versus placebo in reducing adverse LV remodeling in 50 patients with acute anterior ST-segment elevation MI (STEMI) and LVEF of 45% or less [126]. In this trial, the patients were randomly assigned to receive either 20 mg/day of paroxetine or a placebo. Following 3 months of treatment, paroxetine did not affect the recovery of LVEF from baseline. The lack of significant changes in LV end-diastolic volume (LVEDV) (mean difference, +13.4 mL; *p* = 0.30) and LV end-systolic volume (LVESV) (mean difference, +11.4 mL; *p* = 0.13) indicates that paroxetine did not improve LVEF recovery after MI when compared to placebo [126].

The CARE-AMI trial was extended for 1 year to evaluate changes in LVEF from baseline, monitor HF clinical symptoms, and assess major adverse cardiac events [127]. The findings revealed that over the course of 1 year, both the paroxetine and control groups experienced a similar, non-significant improvement in mean LVEF and global longitudinal strain, comparable to the improvements observed in the patients who underwent a 3-month course. Furthermore, no significant differences were observed between the two groups regarding changes in LV dimensions, volumes, or diastolic dysfunction parameters [127]. Overall, the paroxetine therapy, both in the short term and long term, did not lead to any improvement in LVEF following an MI. However, the trial’s reliability is constrained by the small patient sample and the use of less sensitive or different techniques to measure outcomes at the 3-month and 1-year follow-ups. For instance, LV function was evaluated using magnetic resonance imaging at 3 months and echocardiography at 1 year.

From the CARE-AMI study, paroxetine demonstrated a favorable safety profile [126,127]. Since paroxetine extensively distributes throughout the body, especially the central nervous system, the common adverse effects include insomnia, drowsiness, nervousness, loss of appetite, abnormal ejaculation, decreased libido, genital disorders, impotence, asthenia, and constipation [146]. Despite paroxetine’s ability to inhibit GRK2 as an off-target effect [147], its neurological side effects limit its use in HF therapy, driving researchers to design and develop selective small-molecule agents that inhibit GRK2 without these adverse effects. A retrospective cohort study spanning 11 years was conducted to evaluate the relationship between paroxetine treatment and mortality in HF patients. The findings revealed that treatment with paroxetine was linked to an extended survival time, with a 203-day increase at the 3000-day truncation point (*p* = 0.37). Furthermore, paroxetine did not result in a significant reduction in 28-day all-cause mortality among HF patients [148].

A previous study evaluated the efficacy of paroxetine compared to fluoxetine in acute MI patients with depression (AMID) [145]. Patients with AMID (N = 67) were randomized to receive paroxetine (20 mg/day) or fluoxetine (20 mg/day) for the treatment of depression (Table 5). After 2 months, the patients treated with paroxetine exhibited a significant decline in GRK2 levels, recovered autonomic nervous system functions, and an enhancement in LVEF, whereas fluoxetine did not achieve reductions in GRK2 levels or improvements in cardiac performance [145]. Thus, these results imply that the cardioprotective effects of paroxetine were likely independent of its serotonin reuptake blockade activity.

Notably, paroxetine has been demonstrated to possess anti-inflammatory effects across various cell types and tissues [149,150]. However, the molecular mechanisms underlying paroxetine’s anti-inflammatory effects remain unclear, specifically whether they result from the inhibition of serotonin signaling or the inhibition of GRK2 activity. Although paroxetine treatment did not enhance cardiac performance, particularly in terms of increasing LVEF in patients with acute anterior MI (Table 5), the potential pleiotropic anti-inflammatory effects of paroxetine cannot be dismissed. Since colchicine, an anti-inflammatory alkaloid, has demonstrated cardiovascular benefits due to its anti-inflammatory effects [32], low-dose colchicine has been approved as an adjunct therapy to reduce the risk of cardiovascular events in cardiovascular diseases, as indicated in the European Society of Cardiology (ESC) guideline on cardiovascular disease prevention in clinical practice [151].

### 7.3. Clinical Studies of CaMKII Inhibitors

CaMKII is one of the major downstream effectors of β_1_-AR signaling in the heart [52] and plays an important role in cardiac contractility and the development of HF [53]. The upregulation of CaMKII causes cardiac abnormalities including myocardial hypertrophy, myocardial remodeling, arrhythmia, and dilated cardiomyopathy [152]. Thus, the inhibition of CaMKII activity is one of the potential therapeutic targets for the treatment of HF (Table 6). In the last decade, several methods have been extensively used to inhibit CaMKII activity and signaling such as small molecules, peptides, RNA interference, and CRISPR in basic and animal studies with less progress in the clinical trials [152,153]. For instance, hesperidin (a novel CaMKII-δ inhibitor) exerts cardioprotective effects by attenuating myocyte death, myocardial damage, and HF in mice models of ischemia/reperfusion injury [154]. The administration of RA306 (CaMKII-δ/CaMKII-γ dual inhibitor) improved cardiac functions in mice models of dilated cardiomyopathy [155]. These findings suggest that the inhibition of CaMKII exerts beneficial effects as a potential treatment for HF.

NP2O2 is a first-in-class CaMKII-δ inhibitor that was clinically evaluated for the prevention of post-MI in patients after primary percutaneous coronary intervention (PCI) for anterior STEMI and LVEF less than 45% [156]. In this phase II clinical trial, patients were randomized to receive either NP2O2 (1,000 mg/day) or a placebo for 3 months. There was no difference in the primary endpoint as determined by the change in LVESV index from baseline between the placebo and NP2O2 groups (*p* = 0.78). In addition, there was no change in the LVEDV index, LVEF, infarct size, and diastolic function. Despite no difference between the two groups, NP2O2 was well tolerated [156]. Due to NP2O2 not being a potent inhibitor, further clinical studies should investigate other potent CaMKII inhibitors.

## 8. Gene Therapy as a Potential Therapeutic Approach for HF

Gene therapy is gaining recognition as a potential therapeutic strategy for HF, with several cardiac proteins (e.g., AC6 and SERCA2a) and β-ARKct currently being investigated as therapeutic targets [157].

### 8.1. Clinical Studies of AC6 Gene Therapy

Another potential approach for treating HF is the intracoronary delivery of the AC6 gene to failing hearts, where the levels and function of AC6 are reduced (Table 7). The positive effects of AC6 gene transfer were presented in a clinical study in 56 HFrEF patients (NCT00787059) [158]. A single intracoronary administration of adenovirus serotype 5-cytomegalovirus-human AC6 (Ad5-CMV/hAC6) showed a favorable safety profile and notably enhanced LV function, as demonstrated by an increase in LV peak, while exercise capacity remained unchanged. Additionally, hospitalization rates due to HF were reduced by Ad5-CMV-hAC6 compared to the placebo group one year after randomization [158]. However, the sample size was small, indicating the need for further investigations. The FLOURISH phase III clinical trial (NCT03360448) was recently designed to assess the effects of a single intracoronary dose of Ad5-CMV/hAC6 (known as RT-100) or placebo in a group of 536 HFrEF patients with an LVEF of 10-35%. The primary outcome was the reduction in hospitalization rate due to HF from baseline to 12 months [159]. In addition, cardiovascular death, all-cause mortality, HF events, and NYHA functional classification were observed as secondary endpoints. Although the FLOURISH study was granted fast-track designation by the Food and Drug Administration in December 2017, the data are not available.

### 8.2. Clinical Studies of SERCA2a Gene Therapy

SERCA2a, the primary subtype of SERCA found in the heart, plays a crucial role in Ca^2+^ cycling within cardiomyocytes, thereby controlling the contractility and relaxation of the myocardium. In failing hearts, SERCA2a expression and activity are diminished in animal models and humans, which restricts cardiac contraction and ultimately reduces the heart’s ability to pump blood during systole [160]. Enhancing the expression of SERCA2a through genetic manipulation in the heart is considered as one of the promising approaches for the treatment of HF. Adeno-associated virus serotype 1 (AAV1), a cardiotropic vector, has been developed and used in multiple clinical trials, where gene transfer techniques have proven effective in enhancing SERCA2a expression for the treatment of complex conditions including HF [161,162,163,164] (Table 8).

The initial clinical study, CUPID trial (NCT00454818), showed that AAV1/SERCA2a intracoronary infusion had positive efficacy and safety in 39 patients with advanced HF (NYHA class III–IV; LVEF ≤ 30%), as evidenced by improvements in clinical outcomes, including NYHA class, 6 min walk test, peak maximum oxygen consumption (VO_2_ max), NT-proBNP levels, and LVESV, as well as an extended time to clinical events [161]. The patients who received AAV1/SERCA2a experienced a reduction in the frequency of cardiovascular events and the length of cardiovascular-related hospitalizations [161]. The encouraging results from the initial trial prompted the need for larger studies, the CUPID2 trial (NCT01643330), to further evaluate the clinical benefits and safety of AAV1/SERCA2a gene transfer in a greater number of HF patients (NYHA class II–IV; LVEF ≤ 35%; N = 250) [162]. However, AAV1/SERCA2a yielded disappointing results, as it failed to significantly impact the HF clinical progression [162], highlighting the need for additional research. Moreover, the neutral outcomes of the CUPID-2 trial resulted in the early discontinuation of other related studies, including the AGENT-HF trial (NCT01966887) [163] and the SERCA-LVAD trials (NCT00534703) [164], which examined the impact of AAV1/SERCA2a gene delivery on ventricular remodeling in patients with advanced systolic HF and in patients with chronic HF supported by an LV assist device, respectively.

## 9. Limitations

Translating findings from preclinical animal models of HF to human patients presents several limitations. Significant species differences between rodents or other mammals and humans limit the direct application of these models to HF patients. Moreover, many animal models involve a sudden onset of conditions that do not accurately reflect the progressive development of human pathologies associated with HF, often complicated by comorbidities [165]. In addition, HF patients have differences in genetic background, clinical manifestations, and the presence of concomitant conditions. These distinct differences cannot be recapitulated in animal studies. Furthermore, the genetic homogeneity of β-ARs, including downstream effectors in animals, contrasts with the diverse genetic backgrounds in humans. Most studies also use young animals, which fail to replicate the age-related aspects of human HF. The lack of a suitable animal model that fully reflects the complexity and heterogeneity of human HF underscores the necessity of using both small and larger animal HF models to gain a comprehensive understanding of the disease and to investigate therapeutic strategies [166,167].

## 10. Conclusions and Further Directions

Based on the data from previous studies, abnormalities in β-AR regulation via the receptor locus as well as G proteins and targeted effectors have significant impacts affecting β-AR-mediated signal transduction, including biological responses in the pathology of heart diseases. Currently, numerous studies attempt to explain the differences in signal transduction and the regulation of the receptor between the subtypes of β-ARs. The upregulation of β_1_-ARs causes detrimental effects, whereas the stimulation of β_2_-ARs and β_3_-ARs exhibit cardioprotective effects. Hence, the blockade of β_1_-AR with β-blockers is a key pharmacological intervention for HF. Nevertheless, there are limited clinical studies on β_2_-AR agonists and β_3_-AR agonists that have explored their clinical outcomes in patients with HF. The desensitization and downregulation of β-ARs is the pathological hallmark of HF. The degree of HF correlated with a reduction in β_1_-AR density and the upregulation of GRK activity and synthesis, especially GRK2, are found in the failing heart. Thus, the inhibition of GRK2 activity including via β-ARKct gene therapy and synthetic GRK inhibitors represents promising therapeutic strategies for the treatment of HF. However, the effectiveness and clinical outcomes of these GRK2 inhibitors have yet to be evaluated, and initial safety data for these compounds are necessary.

Similar to the regulation of β-ARs at the receptor locus, the changes in the function and level of G proteins can be found in failing hearts. Interestingly, a peptide inhibitor of Gαs protein can suppress β-AR-mediated Gαs protein signaling. In addition, gene therapy targeting the Gαi2 protein has shown beneficial effects for the intervention of arrhythmias. Therefore, targeting β-AR signaling at the G protein level could be considered a promising therapeutic approach for treating HF. At the effector locus of β-AR, AC is one of the essential effectors for β-AR/G protein signaling. However, the specificity of AC isoforms that are related to the pathogenesis of HF is unclear. In addition, the potential therapeutic use of novel compounds selectively inhibiting each AC isoform in the heart is required for further study in animal models of HF. Interestingly, AC6 gene transfer in HF patients showed a significant improvement in cardiac performance. However, the phase III clinical trial with a large number of patients is still under investigation. β-Arrestin-mediated β-AR signaling might initiate signals that are different from Gα protein; hence, β-arrestins are involved in the regulation of β-AR functions in normal and failing hearts. β-Arrestin-biased β-blockers (e.g., carvedilol, metoprolol, and nebivolol) can activate β-arrestin-dependent signaling pathways that are independent of G proteins, thereby enhancing cardioprotection. As a result, β-arrestins have emerged as promising targets for HF therapy.

In the heart, the stimulation of β_1_-AR leads to the activation of CaMKII; however, the upregulation of β_1_-AR-mediated CaMKII activation can cause detrimental effects and the development of HF. Thus, CaMKII inhibition has been an attractive strategy for the treatment of HF. Despite several studies having demonstrated the cardioprotective effects of various types of CaMKII inhibitors in preclinical studies, NP2O2, which is a CaMKII-δ inhibitor, failed to improve cardiac performance in post-MI patients. Even though this inhibitor was well tolerated and safe, we cannot rule out the effects of CaMKII inhibitor in the central nervous system, especially learning and memory. In addition, therapeutic strategies are also challenged by the diversity of CaMKII isoforms and splice variants for the treatment of heart diseases. SERCA2a is one of the potential targets for β-AR/CaMKII signaling in the heart. Up to date, SERCA2a gene therapy has been performed in patients with HFrEF, mostly in phase II clinical trials with a few of patients. The gene therapy of SERCA2a failed to improve the clinical outcome in HF patients and some trials were terminated due to the neutral results. Therefore, there is still a demand for clinical trials with long durations and large patient populations to investigate the effectiveness and safety of gene delivery.

## Figures and Tables

**Table 1:** Genetic polymorphisms of β-ARs affecting heart failure (HF).

β-AR Alleles	Main Findings	Ref.
**β_1_-AR Polymorphisms**		
β_1_-AR-Arg389	Provided a better response to β-blocker therapy in both HF patients and animals compared to β_1_-AR-Gly389	[80,84,85]
	Impaired cardiac functions and led to failing heart in transgenic mice with β_1_-AR-Arg389	[84]
β_1_-AR-Gly389	Expressed a high risk of HF in the East Asian population	[80]
β_1_-AR-Gly49	Had higher level of agonist-induced β_1_-AR downregulation	[86]
β_1_-AR-Gly49	No relationship between the prognosis and risk of HF	[80]
β_1_-AR-Ser49	Required a higher dose of β-blockers in the management of HF compared to β_1_-AR-Gly49	[87]
**β_2_-AR Polymorphisms**		
β_2_-AR-Glu27	Increased risk of insulin resistance in HF patients	[88]
β_2_-AR-Ile164	Reduced efficiency of β_2_-AR signaling and responses in HF patients	[89]

**Abbreviations**: Arg, arginine; Gly, glycine; HF, heart failure; Ile, isoleucine; Ser, serine.

**Table 2:** Inhibition of GRK2 activity in HF.

Study Models	Main Findings	Ref.
Cardiac-specific β-ARKct overexpression in HF mice	▪ Prevented the progression of cardiomyopathy ▪ Restored impaired heart function	[121]
Cardiac-specific β-ARKct overexpression in HF mice	▪ Increased cardiac contractility ▪ Improved cardiac functions	[115]
Cardiac-specific β-ARKct overexpression in HF mice	▪ Prolonged survival ▪ Augmented β-AR antagonist therapy	[122]
GRK2 ablation in MI mice	▪ Increased survival rate ▪ Enhanced cardiac performance ▪ Prevented cardiac remodeling	[123]

**Abbreviations**: HF, heart failure; MI, myocardial infarction.

**Table 3:** Effects of β-arrestin-biased ligands for β-ARs.

β-Arrestin-Biased Ligands	Study Models	Main Findings	Ref.
Alprenolol Carvedilol	HEK-293 cells and mice	▪ Stimulated β-arrestin-mediated EGFR transactivation and downstream ERK activation	[137]
Carvedilol Propranolol	Rat hippocampal neurons	▪ Inhibited G protein signaling ▪ Mediated neuronal Ca^2+^ signaling through β-arrestin2 and ERK1/2	[138]
Carvedilol	β_2_-AR-expressing HEK-293 cells	▪ Acted as β-arrestin biased ligand for the activation of β-arrestin-dependent ERK1/2 signaling	[45]
Metoprolol	Cardiac myocytes and GRK5/β-arrestin2-knock out mice	▪ Induced cardiac fibrosis in a G protein-independent and GRK5/β-arrestin2-dependent manner	[139]
Nebivolol	Mouse embryonic fibroblasts and cardiac myocytes	▪ Suppressed ERK activation by the knockout of β-arrestin1/2	[140]

**Abbreviations**: EGFR, epidermal growth factor receptor; ERK, extracellular-signal-regulated kinase; GRK5, G protein-coupled receptor kinase 5.

**Table 4:** Clinical studies of β_3_-AR agonist.

Drugs	Phase	Participants	Treatment	Main Findings	Ref.
Mirabegron (Beta3-LVH trial)	II	Patients who had LV hypertrophy with or without HF symptoms (NYHA I-II) (N = 296)	Mirabegron 50 mg/day or placebo **Duration:** 12 months	▪ Increased LV mass index ▪ Decreased LV diastolic function	[142]
Mirabegron (BEAT-HF trial)	II	HFrEF patients with LVEF < 40% and NYHA II-III (N = 70)	Mirabegron 300 mg/day or placebo **Duration:** 6 months	▪ No change in LVEF ▪ No change in volumetric parameters, physical capacity, and electrocardiogram	[143]
Mirabegron (BEAT-HF-II trial)	II/III	HFrEF patients with NYHA III–IV, LVEF < 35%, and increased NT-proBNP levels (N = 22)	Mirabegron 300 mg/day or placebo **Duration:** 1 week	▪ Improved cardiac performance by increased cardiac index and reduced PVR ▪ No change in blood pressure, heart rate, and systemic vascular resistance	[73]
Mirabegron (SPHERE-HF trial)	II	Patients with combined pre- and post-capillary pulmonary hypertension (CpcPH) associated with symptomatic HF (N = 80)	Mirabegron 200 mg/day or placebo **Duration:** 16 weeks	▪ No change in PVR ▪ Increased right ventricular ejection fraction ▪ No change in hemodynamic parameters and functional class	[144]

**Abbreviations**: HF, heart failure; HFrEF, heart failure with reduced ejection fraction; LV, left ventricular; LVEF, left ventricular ejection fraction; NYHA, New York Heart Association functional class; PVR, pulmonary vascular resistance.

**Table 5:** Clinical studies of GRK2 inhibitor.

Drug	Phase	Participants	Treatment	Main Findings	Ref.
Paroxetine (CARE-AMI trial)	II	Patients with acute anterior MI with LVEF ≤ 45% (N = 50)	Paroxetine 20 mg/day or placebo **Duration:** 3 months	▪ No difference in LVEF recovery ▪ No change in LVEDV and LVESV ▪ No improvement in LVEF after MI	[126]
	II	Patients with acute anterior MI with LVEF ≤ 45% (N = 50)	Paroxetine 20 mg/day or placebo **Duration:** 1 year	▪ No change in LV dimensions and volumes ▪ No difference in the parameters of diastolic dysfunction ▪ No effect on LVEF recovery	[127]
Paroxetine and fluoxetine	II	Acute MI patients with depression (N = 67)	Paroxetine 20 mg/day or fluoxetine 20 mg/day **Duration:** 2 months	**Paroxetine:** ▪ Decreased GRK2 levels ▪ Improved cardiac functions ▪ Normalized ANS functions **Fluoxetine:** ▪ Normalized ANS functions	[145]

**Abbreviations**: ANS, autonomic nervous system; LVEF, left ventricular ejection fraction; LVEDV, left ventricular end-diastolic volume; LVESV, left ventricular end-systolic volume; MI, myocardial infarction.

**Table 6:** Clinical studies of CaMKII inhibitor.

Drug	Phase	Participants	Treatment	Main Findings	Ref.
NP2O2	II	Patients after PCI for anterior STEMI with LVEF ≤ 45% (N = 147)	NP2O2 at 1000 mg/day or placebo **Duration:** 3 months	▪ No difference in the LVESV index ▪ No change in the LVEDV index, LVEF, infarct size, and diastolic function ▪ Well-tolerated	[156]

**Abbreviations**: PCI, percutaneous coronary intervention; LVEDV, left ventricular end-diastolic volume; LVESV, left ventricular end-systolic volume; STEMI, ST-segment elevation myocardial infarction.

**Table 7:** Clinical studies of AC6 gene delivery.

Drug	Phase	Participants	Treatment	Main Findings	Ref.
Ad5.hAC6	II	Patients with symptomatic HF and LVEF ≤ 40% (N = 56)	Ad5.hAC6 (3.2 × 10^9^ to 10^12^ particles) or placebo, Intracoronary **Duration:** 1 year	▪ Increased EF and LV peak -dP/dt ▪ No change in exercise duration ▪ Safe and did not increase arrhythmias	[158]
Ad5.hAC6 (or RT-100) (FLOURISH trial)	III	HFrEF patients with LVEF 10–35% (N = 536)	Ad5.hAC6 (10^12^ particles) or placebo, Intracoronary **Duration:** 1 year	▪ Not available	[159]

**Abbreviations**: HF, heart failure; Ad5.hAC6, adenovirus 5 encoding adenylyl cyclase 6; LVEF, left ventricular ejection fraction.

**Table 8:** Clinical studies of SERCA2a gene delivery.

Drug	Phase	Participants	Treatment	Main Findings	Ref.
AAV1/ SERCA2a (CUPID trial)	II	Patients with advanced HF (NYHA class III-IV; LVEF ≤ 30%) (N = 39)	AAV1/SERCA2a (1 × 10^13^ particles) or placebo, Intracoronary **Duration:** 1 year	▪ Improved HF clinical outcome ▪ Increased time to HF clinical events ▪ Decreased frequency of CV events and duration of CV hospitalization	[161]
AAV1/ SERCA2a (CUPID2 trial)	II	Patients with HF (NYHA class II-IV; LVEF ≤ 35%) (N = 250)	AAV1/SERCA2a (1 × 10^13^ particles) or placebo, Intracoronary **Duration:** 1 year	▪ Did not improve clinical outcomes ▪ No change in time to recurrent CV events	[162]
AAV1/ SERCA2a (AGENT-HF trial)	II	Patients with advanced HF (NYHA class II-IV; LVEF ≤ 35%) (N = 9)	AAV1/SERCA2a (1 × 10^13^ particles) or placebo, Intracoronary **Duration:** 6 months	▪ Improved LVESV (non-significance) ▪ The trial was terminated due to the neutral results from the CUPID2	[163]
AAV1/ SERCA2a (SERCA-LVAD trial)	II	Patients with chronic HF and implanted with an LV assist device (N = 5)	AAV1/SERCA2a (1 × 10^13^ particles) or placebo, Intracoronary **Duration:** 3 years	▪ Viral DNA was delivered into a failing heart, although overall levels of detectable transgene DNA remained low ▪ The trial was terminated due to the neutral results from the CUPID2	[164]

**Abbreviations**: AAV1, adeno-associated virus serotype 1; CV, cardiovascular; LVEF, left ventricular ejection fraction; LVESV, left ventricular end-systolic volume; NYHA, New York Heart Association functional class.

## Data Availability

This is a Review article and there are no new data in the manuscript.
